# Supplementary material for: Burden, risk factors, and clinical outcomes of pediatric malaria in Nigeria: A systematic review and meta-analysis protocol
Source: PLoS One. 2026 Mar 13;21(3):e0336091. doi: 10.1371/journal.pone.0336091 (PMC12987443; doi:10.1371/journal.pone.0336091)
Supplement: S2 File — a. The review work is self-funded. b. Sponsor: All the authors contributed to sponsoring the project. c. Guarantor of the review: ORI. (DOCX) [file pone.0336091.s002.docx]

**Search string for the study**

| Database | Search terms |
| --- | --- |
| PubMed | ("Nigeria"[Mesh] OR “Nigeria” OR “Nigerian”)  AND  ("Child"[Mesh] OR "Infant"[Mesh] OR "Adolescent"[Mesh] OR "Pediatrics"[Mesh] OR "Infant, Newborn"[Mesh] OR child*[tiab] OR infant*[tiab] OR adolescent*[tiab] OR pediatric*[tiab] OR paediatric*[tiab] OR newborn*[tiab] OR neonate*[tiab] OR "school-age"[tiab] OR "under-five"[tiab] OR "under five"[tiab] OR "young children"[tiab])  AND  ( "Malaria"[Mesh] OR "Malaria, Falciparum"[Mesh] OR "Malaria, Vivax"[Mesh] OR "Plasmodium falciparum"[Mesh] OR malaria[tiab] OR "severe malaria"[tiab] OR "uncomplicated malaria"[tiab] OR "Plasmodium falciparum"[tiab] OR "Plasmodium infection*"[tiab] OR "malaria infection"[tiab] OR "malaria burden"[tiab] OR "malaria morbidity"[tiab] OR "malaria mortality"[tiab] OR "malaria hospitalization"[tiab])  AND  ("Risk Factors"[Mesh] OR "Socioeconomic Factors"[Mesh] OR "Sex Factors"[Mesh] OR "Epidemiology"[Mesh] OR "Disease Susceptibility"[Mesh] OR "Social Determinants of Health"[Mesh] OR determinant*[tiab] OR "associated factor*"[tiab] OR "predictor*"[tiab] OR "correlate*"[tiab] OR "socioeconomic status"[tiab] OR "education level"[tiab] OR "household income"[tiab] OR "parental occupation"[tiab] OR "sex difference*"[tiab] OR "gender difference*"[tiab] OR "clinical outcome*"[tiab] OR "treatment outcome*"[tiab] OR mortality[tiab] OR death*[tiab] OR fatal*[tiab] OR discharge[tiab] OR complication*[tiab] OR "neurological sequelae"[tiab]  OR hospitalization[tiab] OR "disease severity"[tiab] OR "case fatality"[tiab])  AND  ("cross-sectional studies"[Mesh] OR "cohort studies"[Mesh] OR "case-control studies"[Mesh] OR "observational study"[Publication Type] OR "interventional studies"[Mesh] OR "clinical trials "[Mesh] OR "longitudinal studies"[Mesh] OR "epidemiologic studies"[Mesh] OR "survey"[tiab] OR "prevalence"[tiab] OR "incidence"[tiab] OR "retrospective"[tiab] OR "prospective"[tiab]) |
| Web of Science | TS=(Nigeria OR Nigerian OR)  AND  TS=(child* OR infant* OR adolescent* OR youth* OR newborn* OR neonate*  OR pediatric* OR paediatric* OR "school-age" OR "school aged"  OR "under-five" OR "under five" OR "young children" OR "adolescence"  OR "pre-school" OR "preschool" OR "toddler*")  AND  TS=("malaria" OR "severe malaria" OR "uncomplicated malaria"  OR "Plasmodium falciparum" OR "Plasmodium infection*"  OR "falciparum malaria" OR "childhood malaria"  OR "malaria infection" OR "malaria cases"  OR "malaria burden" OR "malaria morbidity"  OR "malaria mortality" OR "malaria hospitalization"  OR "malaria admission" OR "malaria prevalence" OR "malaria incidence")  AND  TS=("risk factor*" OR "associated factor*" OR determinant* OR "predictor*"  OR "correlate*" OR "socioeconomic factor*" OR "socioeconomic status"  OR "education level" OR "household income" OR "parental occupation"  OR "living conditions" OR "environmental factor*"  OR "sex factor*" OR "gender difference*" OR "sex difference*"  OR "demographic factor*" OR "health disparity*"  OR "health inequality" OR "disease susceptibility"  OR "social determinant*" OR "climatic factor*" OR "rainfall"  OR "seasonality" OR "clinical outcome*" OR "treatment outcome*"  OR mortality OR death* OR fatal* OR discharge OR complication*  OR "neurological sequelae" OR hospitalization OR "disease severity"  OR "case fatality" OR morbidity OR "hospital outcome*"  OR "disease outcome*" OR "public health implication*")  AND  TS=("cross-sectional" OR "cohort" OR "case-control" OR "observational" OR “interventional studies” OR “clinical trials”  OR "epidemiologic" OR "survey" OR "retrospective" OR "prospective"  OR "longitudinal" OR "prevalence" OR "incidence" OR "hospital-based"  OR "community-based" OR "multicenter" OR "multi-centre") |
|  | <https://www.webofscience.com/wos/woscc/summary/f3d2b6c4-1a08-4793-8df8-6488e3dcedcc-0181604609/relevance/1> |
| Scopus | TITLE-ABS-KEY(Nigeria OR Nigerian OR "West Africa" OR "Sub-Saharan Africa")  AND  TITLE-ABS-KEY(child* OR infant* OR adolescent* OR youth* OR newborn* OR neonate*  OR pediatric* OR paediatric* OR "school-age" OR "school aged"  OR "under-five" OR "under five" OR "young children" OR "pre-school" OR "preschool"  OR toddler*)  AND  TITLE-ABS-KEY("malaria" OR "severe malaria" OR "uncomplicated malaria"  OR "falciparum malaria" OR "Plasmodium falciparum" OR "Plasmodium infection*"  OR "malaria infection" OR "childhood malaria" OR "malaria cases"  OR "malaria burden" OR "malaria morbidity" OR "malaria mortality"  OR "malaria hospitalization" OR "malaria admission"  OR "malaria prevalence" OR "malaria incidence")  AND  TITLE-ABS-KEY("risk factor*" OR "associated factor*" OR determinant* OR "predictor*"  OR "correlate*" OR "socioeconomic factor*" OR "socioeconomic status"  OR "education level" OR "household income" OR "parental occupation"  OR "living conditions" OR "environmental factor*" OR "climatic factor*"  OR rainfall OR "seasonality" OR "sex factor*" OR "gender difference*"  OR "sex difference*" OR "demographic factor*" OR "health disparity*"  OR "health inequality" OR "disease susceptibility"  OR "social determinant*" OR "clinical outcome*" OR "treatment outcome*"  OR mortality OR death* OR fatal* OR discharge OR complication*  OR "neurological sequelae" OR hospitalization OR "disease severity"  OR "case fatality" OR morbidity OR "hospital outcome*" OR "disease outcome*")  AND  TITLE-ABS-KEY("cross-sectional" OR "cohort" OR "case-control" OR "observational"  OR "epidemiologic" OR "survey" OR "retrospective" OR "prospective"  OR "longitudinal" OR "prevalence" OR "incidence"  OR "hospital-based" OR "community-based" OR "multicenter" OR "multi-centre") |
| EBSCOhost | (TX Nigeria OR TX Nigerian OR)  AND  (TX child* OR TX infant* OR TX adolescent* OR TX youth* OR TX newborn*  OR TX neonate* OR TX pediatric* OR TX paediatric* OR TX "school-age"  OR TX "school aged" OR TX "under-five" OR TX "under five"  OR TX "young children" OR TX "pre-school" OR TX preschool OR TX toddler*)  AND  (TX malaria OR TX "severe malaria" OR TX "uncomplicated malaria"  OR TX "falciparum malaria" OR TX "Plasmodium falciparum"  OR TX "Plasmodium infection*" OR TX "malaria infection"  OR TX "childhood malaria" OR TX "malaria cases" OR TX "malaria burden"  OR TX "malaria morbidity" OR TX "malaria mortality"  OR TX "malaria hospitalization" OR TX "malaria admission"  OR TX "malaria prevalence" OR TX "malaria incidence")  AND  (TX "risk factor*" OR TX "associated factor*" OR TX determinant*  OR TX predictor* OR TX correlate* OR TX "socioeconomic factor*"  OR TX "socioeconomic status" OR TX "education level"  OR TX "household income" OR TX "parental occupation"  OR TX "living conditions" OR TX "environmental factor*"  OR TX "climatic factor*" OR TX rainfall OR TX seasonality  OR TX "sex factor*" OR TX "gender difference*" OR TX "sex difference*"  OR TX "demographic factor*" OR TX "health disparity*"  OR TX "health inequality" OR TX "disease susceptibility"  OR TX "social determinant*" OR TX "clinical outcome*"  OR TX "treatment outcome*" OR TX mortality OR TX death*  OR TX fatal* OR TX discharge OR TX complication*  OR TX "neurological sequelae" OR TX hospitalization  OR TX "disease severity" OR TX "case fatality"  OR TX morbidity OR TX "hospital outcome*" OR TX "disease outcome*")  AND  (TX "cross-sectional" OR TX "cohort" OR TX "case-control" OR TX observational  OR TX epidemiologic OR TX survey OR TX retrospective  OR TX prospective OR TX longitudinal OR TX prevalence  OR TX incidence OR TX "hospital-based" OR TX "community-based"  OR TX multicenter OR TX "multi-centre" OR TX "registry study") |
| EMBASE (Elsevier / Ovid Platform) | ('nigeria'/exp OR Nigeria:ti,ab OR Nigerian:ti,ab)  AND  ('child'/exp OR 'infant'/exp OR 'adolescent'/exp OR 'pediatrics'/exp  OR child*:ti,ab OR infant*:ti,ab OR adolescent*:ti,ab OR pediatric*:ti,ab  OR paediatric*:ti,ab OR newborn*:ti,ab OR neonate*:ti,ab OR "school-age":ti,ab  OR "school aged":ti,ab OR "under-five":ti,ab OR "under five":ti,ab  OR "young children":ti,ab OR "pre-school":ti,ab OR preschool:ti,ab OR toddler*:ti,ab)  AND  ('malaria'/exp OR 'falciparum malaria'/exp OR 'plasmodium falciparum'/exp  OR malaria:ti,ab OR "severe malaria":ti,ab OR "uncomplicated malaria":ti,ab  OR "falciparum malaria":ti,ab OR "Plasmodium infection*":ti,ab  OR "malaria infection":ti,ab OR "childhood malaria":ti,ab  OR "malaria burden":ti,ab OR "malaria morbidity":ti,ab  OR "malaria mortality":ti,ab OR "malaria hospitalization":ti,ab  OR "malaria prevalence":ti,ab OR "malaria incidence":ti,ab)  AND  ('risk factor'/exp OR 'socioeconomic factor'/exp OR 'sex factor'/exp  OR 'health inequality'/exp OR 'disease susceptibility'/exp  OR 'social determinant of health'/exp OR determinant*:ti,ab  OR "associated factor*":ti,ab OR predictor*:ti,ab OR correlate*:ti,ab  OR "socioeconomic status":ti,ab OR "education level":ti,ab  OR "household income":ti,ab OR "parental occupation":ti,ab  OR "living conditions":ti,ab OR "environmental factor*":ti,ab  OR "climatic factor*":ti,ab OR rainfall:ti,ab OR seasonality:ti,ab  OR "sex factor*":ti,ab OR "gender difference*":ti,ab OR "sex difference*":ti,ab  OR "demographic factor*":ti,ab OR "health disparity*":ti,ab  OR "disease susceptibility":ti,ab OR "social determinant*":ti,ab  OR "clinical outcome*":ti,ab OR "treatment outcome*":ti,ab  OR mortality:ti,ab OR death*:ti,ab OR fatal*:ti,ab OR discharge:ti,ab  OR complication*:ti,ab OR "neurological sequelae":ti,ab  OR hospitalization:ti,ab OR "disease severity":ti,ab OR "case fatality":ti,ab  OR morbidity:ti,ab OR "hospital outcome*":ti,ab OR "disease outcome*":ti,ab  OR "public health implication*":ti,ab)  AND  ('cross-sectional study'/exp OR 'cohort study'/exp OR 'case control study'/exp  OR 'observational study'/exp OR 'epidemiology'/exp OR "cross-sectional":ti,ab  OR "cohort":ti,ab OR "case-control":ti,ab OR "observational":ti,ab  OR "survey":ti,ab OR "retrospective":ti,ab OR "prospective":ti,ab  OR "longitudinal":ti,ab OR "prevalence":ti,ab OR "incidence":ti,ab  OR "hospital-based":ti,ab OR "community-based":ti,ab  OR "multicenter":ti,ab OR "multi-centre":ti,ab OR "registry study":ti,ab) |
| Google Scholar | ("pediatric malaria" OR "paediatric malaria" OR "childhood malaria" OR "malaria in children" OR "malaria among children" OR "malaria in infants" OR "severe malaria" OR "uncomplicated malaria" OR "falciparum malaria" OR "Plasmodium falciparum infection")  AND ("Nigeria" OR "Nigerian" OR "West Africa" OR "Sub-Saharan Africa")  AND ("risk factors" OR "associated factors" OR determinants OR "socioeconomic status" OR "education level" OR "household income" OR "parental occupation" OR "living conditions" OR "environmental factors" OR "seasonality" OR "rainfall" OR "sex differences" OR "gender differences" OR "clinical outcomes" OR "treatment outcomes" OR complications OR hospitalization OR mortality OR morbidity OR "case fatality" OR "neurological sequelae")  AND ("cross-sectional study" OR "cohort study" OR "case-control study" OR "observational study" OR "epidemiologic study" OR "prevalence" OR "incidence" OR "community-based" OR "hospital-based") |
