# Supplementary material for: Burden, risk factors, and clinical outcomes of pediatric malaria in Nigeria: A systematic review and meta-analysis protocol
Source: PLoS One. 2026 Mar 13;21(3):e0336091. doi: 10.1371/journal.pone.0336091 (PMC12987443; doi:10.1371/journal.pone.0336091)
Supplement: Attachment — Submitted filename: Rebuttal_letter_resubmission_03.pdf [file pone.0336091.s003.pdf]

The Editor,
PLOS One,
Dear Sir/Ma

Re: PLOS ONE Decision: PONE-D-25-55024 - [EMID:46cf7d54569fa28f]

We appreciate the prompt feedback and comments on our earlier submitted manuscript entitled "Burden, Risk Factors, and Clinical Outcomes of Pediatric Malaria in Nigeria: A Systematic Review and Meta-Analysis Protocol".

We have seen revised the manuscript as suggested and find below the changes to the manuscript and where the changes are made.

#Comments

"In the case of your submission, we note that language restrictions are imposed on the literature searches without adequate justification. There is increasing recognition that exclusion of studies published in other languages can increase the risk of bias in systematic reviews. We feel that any restrictions should be justified based on scientific reasons rather than resource constraints. As a result, we have concerns about the comprehensiveness of the review and the validity of the conclusions"

Authors' response

We appreciate the reviewer's observation regarding the language restriction in our literature search. We acknowledge that excluding non-English studies may potentially introduce language bias. However, our decision to include only English-language publications was based on scientific and methodological considerations, rather than resource constraints. First, English is the official language of Nigeria, including publications for Nigerian medical journals and institutions. Second, the Nigerian health research and academic system primarily disseminates findings in English.

Third, this study focused on pediatric malaria in Nigeria, where the vast majority of published epidemiological and clinical studies are reported in English.

However, to address the reviewer's concern transparently, we have revised the Methods section to include studies published in any language. The methods section has been updated in the 'Eligibility Criteria' and 'Search Strategy' sections of this manuscript.

Eligibity criteria

Study design (S): This review will include quantitative observational and interventional studies, including cross-sectional, cohort, case-control, and longitudinal studies, conducted in Nigeria and published in any language. Non-English studies will be translated using professional translation tools, Google Translate (academic mode) and Microsoft Translator. The translated studies will be verified by native-language speaker to ensure accuracy during data extraction (Line 105-109).

Search strategy

There will be no language restrictions during the literature search. All relevant studies, irrespective of language, will be included. Non-English studies identified during the screening will be translated to English before data extraction to minimize selection and translation bias (Lines 138-140).

#Comments

As a result of these concerns, we cannot consider the manuscript for publication at PLOS ONE at this time. However, if you are able to address the concerns discussed above, we may be able to consider a revised version of the manuscript. This would however require a substantial amount of work and thus, would be considered as a new submission. If you are able to revise the manuscript to respond to the concerns noted above, please refer to the original submission in the covering

letter and accompany the submission by a rebuttal letter that responds to the comments made on the original manuscript.

Authors' response

This amendment enhances the inclusiveness, reproducibility, and validity of our systematic review, in alignment with PRISMA and Cochrane guidance and we have included a rebuttal letter as suggested

#Other comments

Please update your Cover Letter to indicate whether you wish to have the manuscript considered as a Study Protocol, and indicate whether data extraction has been completed.

Authors' response

Though the submission title indicated this is a protocol for a systematic review and meta-analysis, this has been explicitly stated in the cover letter as advised. In addition, the third to the last paragraph (cover letter) indicated the stage of the development, which is currently at literature search, screening and data extraction is yet to be started.

#Other comments

Please ensure that your Methods section reports the status and timeline of the study, including an estimate of when A) record screening will be completed, B) data extraction will be completed, and C) results are expected. If any of these stages have already been completed, this should be indicated in the Methods section.

Authors' response

This request was addressed at the last ammendement. For the purpose of clarification, this is included in the Status and timeline of the study subsection of the methods (line 208-212) and highlighted in the tracked version

Your Sincerely,

Olayinka Ibrahim
Corresponding Author
